# The Perineuronal ‘Safety’ Net? Perineuronal Net Abnormalities in Neurological Disorders

**DOI:** 10.3389/fnmol.2018.00270

**Published:** 2018-08-03

**Authors:** Teresa H. Wen, Devin K. Binder, Iryna M. Ethell, Khaleel A. Razak

**Affiliations:** ^1^Neuroscience Graduate Program, University of California, Riverside, Riverside, CA, United States; ^2^Division of Biomedical Sciences, School of Medicine, University of California, Riverside, Riverside, CA, United States; ^3^Psychology Graduate Program, Department of Psychology, University of California, Riverside, Riverside, CA, United States

**Keywords:** perineuronal nets, extracellular matrix, interneurons, excitation/inhibition balance, sensory cortex, Fragile X Syndrome, autism

## Abstract

Perineuronal nets (PNN) are extracellular matrix (ECM) assemblies that preferentially ensheath parvalbumin (PV) expressing interneurons. Converging evidence indicates that PV cells and PNN are impaired in a variety of neurological disorders. PNN development and maintenance is necessary for a number of processes within the CNS, including regulation of GABAergic cell function, protection of neurons from oxidative stress, and closure of developmental critical period plasticity windows. Understanding PNN functions may be essential for characterizing the mechanisms of altered cortical excitability observed in neurodegenerative and neurodevelopmental disorders. Indeed, PNN abnormalities have been observed in post-mortem brain tissues of patients with schizophrenia and Alzheimer’s disease. There is impaired development of PNNs and enhanced activity of its key regulator matrix metalloproteinase-9 (MMP-9) in Fragile X Syndrome, a common genetic cause of autism. MMP-9, a protease that cleaves ECM, is differentially regulated in a number of these disorders. Despite this, few studies have addressed the interactions between PNN expression, MMP-9 activity and neuronal excitability. In this review, we highlight the current evidence for PNN abnormalities in CNS disorders associated with altered network function and MMP-9 levels, emphasizing the need for future work targeting PNNs in pathophysiology and therapeutic treatment of neurological disorders.

## Introduction

**FIGURE 1:**
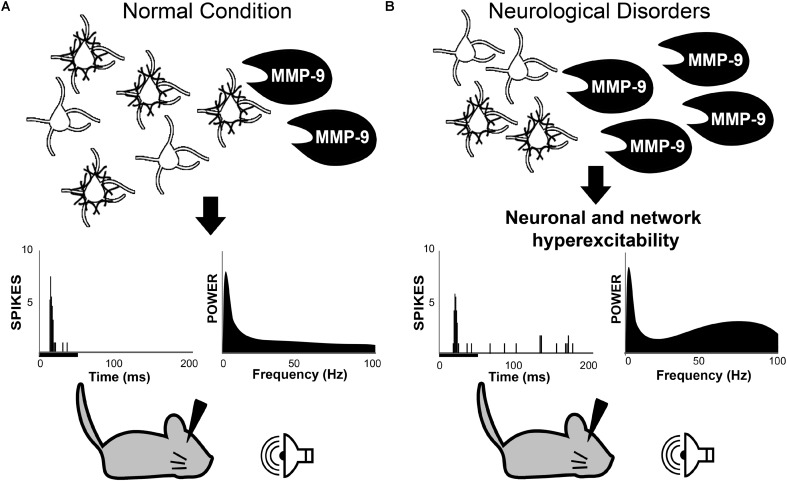
Working hypothesis in which MMP-9 is necessary for normal PV cell function and consequently normal neuronal and network excitability **(A)**. Top row: schematic of PV/PNN cells and cleavage of PNN by MMP-9. Middle row: single unit responses (each vertical line is an action potential in the post-stimulus time histogram) to sound stimulus (black bar under the abscissa) and spectral power of baseline EEGs. **(B)** Increased MMP-9 levels in disorders such as Fragile X Syndrome cause altered PNN formation/composition around PV cells. This may contribute to increased response to sensory stimuli and enhanced gamma power in baseline EEGs. The review argues that multiple neurological and neurodevelopmental disorders may include altered PV/PNN/MMP-9 interactions leading to circuit dysfunction. PV, parvalbumin; PNN, perineuronal nets; MMP-9, matrix metalloproteinase-9.

First described by Golgi and Cajal (Golgi, 1893; Ramón and Cajal, 1897), PNNs are assemblies of ECM proteins, which form net-like structures around neurons (Brauer et al., 1982, 1984). While PNNs were first identified in the late 1800s, much of our current knowledge about PNN structure and function can be attributed to a renewed interest in PNNs almost a century later (Brückner et al., 1993; Celio and Blümcke, 1994; Celio et al., 1998). Since then, PNNs have been implicated in a wide range of functions including neuroprotection, regulation of neural activity and experience-dependent plasticity. Excellent reviews have been written about the role of PNNs in brain development (Berardi et al., 2004; Hensch, 2005; Karetko and Skangiel-Kramska, 2009; Wang and Fawcett, 2012). Recently, increasing evidence suggests that PNNs are altered in various neurodevelopmental and neurodegenerative disorders associated with changes in brain activity (De Luca and Papa, 2016; Sorg et al., 2016; Härtig et al., 2016). In parallel, a number of these CNS diseases exhibit dysregulated expression of enzymes necessary for PNN cleavage and reorganization (Backstrom et al., 1996; Lorenzl et al., 2003; Rybakowski et al., 2009; Kim et al., 2009; Zhang et al., 2011; Yamamori et al., 2013; Liu et al., 2014; Rankin-Gee et al., 2015; Lepeta et al., 2017). However, the role of PNNs in neural and network excitability is only beginning to be understood (Balmer, 2016; Murase et al., 2017; Wen et al., 2017). Based on evidence from recent studies in the mouse model of FXS, we propose a novel working hypothesis in which MMP-9 mediated cleavage and/or reorganization of PNN structures around PV-expressing cells contributes to abnormal PV cell development and cortical hyperexcitability (**Figure 1**). Using this hypothesis as a scaffold, we bring together evidence from studies of schizophrenia, AD, and epilepsy to suggest that systematic studies of the interplay between MMP-9, PNN and PV cells may be key to understanding mechanisms of multiple CNS disorders at the cellular and circuit levels.

## PNN Structure and Developmental Regulation of its Components

**FIGURE 2:**
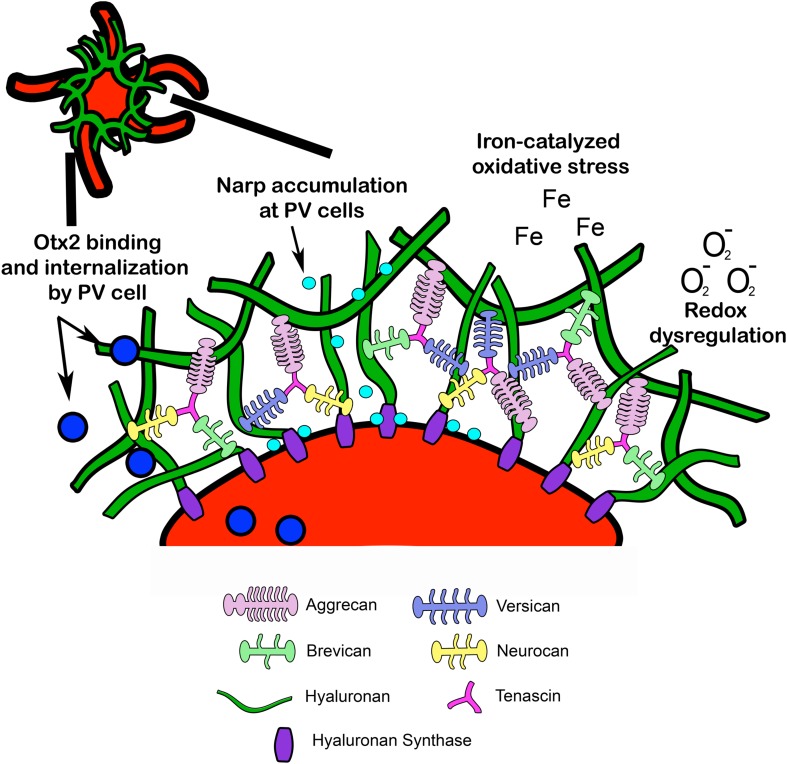
Structural composition of PNN. Lecticans are attached to a hyaluronan backbone via link proteins such as cartilage link protein and hyaluronan link proteins (*not pictured*). Hyaluronan synthase contributes to hyaluronan synthesis and anchors hyaluronan to the cell membrane. Tenascin promotes crosslinking of lecticans and helps maintain PNN structural integrity. PNN around PV cells may provide protection from redox dysregulation and oxidative stressors such as superoxide free radicals (O_2_^-^) and metal ions (Fe). In addition, PNNs may facilitate PV maturation through internalization of Otx2 homeoproteins and regulate PV cell excitability through accumulation of the immediate early gene and synaptic scaling molecule *Narp* (see *Fragile X Syndrome*). Narp, neuronal activity-regulated pentraxin; Otx2, orthodenticle homeoprotein 2.

Perineuronal nets are specialized assemblies of ECM proteins which ensheath neurons and proximal dendrites (Celio et al., 1998). PNNs are composed of CSPGs, tenascin, and link proteins, which facilitate binding to a hyaluronan backbone (Yamaguchi, 1999) (**Figure 2**). Developmental regulation of ECM proteins, which make up PNNs, may underlie PNN formation, composition, and function (Köppe et al., 1997). The developmental changes in major PNN proteins are discussed in the following sections:

### Chondroitin Sulfate Proteoglycans

Chondroitin sulfate proteoglycans consist of a core protein and varying amounts of chondroitin sulfate chains covalently bound to a central region of the protein. These chondroitin sulfate side chains are a type of GAG polysaccharide consisting of disaccharide blocks of *N*-acetylgalactosamine and glucuronic acid (Sherman and Back, 2007). Various CSPGs exist in the ECM throughout the body, but the lectican family of CSPGs is most prominent in PNNs of the CNS. These include aggrecan, neurocan, versican, and brevican, which vary in core protein size and the number of GAG side chains (Yamaguchi, 1999).

Although lecticans are developmentally regulated, their function in CNS development remains unclear. A developmental upregulation of aggrecan and brevican, which peak in adulthood, was reported in rat brain (Milev et al., 1998; Brückner et al., 2000; Popp et al., 2003). Versican consists of two different alpha and beta isoforms, which are differentially expressed during development. The expression of alpha isoform was low during embryonic development, first and second postnatal weeks, and then increased steadily until around 3.5 months of age (Bignami et al., 1993b). In contrast, the expression of beta isoform increased 2–3 fold from embryonic day (E) 14 until birth and then decreased in adulthood. Neurocan expression also increased between E14 and postnatal day (P) 3 and then decreased during early-late adolescence and into adulthood (Meyer-Puttlitz et al., 1996; Miller et al., 1996; Milev et al., 1998; Brückner et al., 2000). Studies of postmortem human tissues also suggest developmental changes in lectican levels in human CNS. For example, high levels of brevican mRNA expression were detected in postmortem human cortex during early development until 8 years of age, and the expression levels were lower in the adult human brain (Gary et al., 2000). These developmental changes in lectican expression levels suggest that the PNNs themselves may also be developmentally regulated, underlying maturation of circuit function (Köppe et al., 1997). However, the functional impact of developmental changes in PNN lectican composition is unknown.

### Tenascins and Hyaluronan

Tenascins and hyaluronan are also part of PNN structure. Tenascins are fibrous, matricellular proteins, which bind to the C-terminal domain of lecticans (Aspberg et al., 1997; Day et al., 2004). Hyaluronan is an unsulfated GAG that is synthesized at the cell surface by the membrane-bound enzymes called HAS and protrudes through the plasma membrane into the extracellular space (Fraser et al., 1997). Hyaluronan binds to other ECM molecules via N-terminal hyaluronan binding domains, including lecticans and link proteins such as cartilage link protein, Ctrl1, and brain-derived link proteins Bral1 and Bral2 (Frischknecht and Seidenbecher, 2008). This complex of hyaluronan with ECM proteins forms the basis for PNN structure. Similar to lecticans, tenascin and hyaluronan are also developmentally regulated. In the mouse cortex, tenascin immunolabeling is first detected around E16 (Sheppard et al., 1991) and tenascin levels increase after birth until the third postnatal week (Brückner et al., 2000). By week 4, tenascin expression is reduced to adult levels (Brückner et al., 2000). Hyaluronan expression precedes the detection of both tenascin and lecticans, and is first observed near the notochord in E11-12 rat embryos (Delpech and Delpech, 1984; Bignami et al., 1993a). Hyaluronan is expressed during postnatal development and adulthood but peaks during embryonic development (Bignami et al., 1993a).

While lecticans, tenascins, and hyaluronan are expressed during embryonic development, the classic net-like structure of PNNs is not typically seen until later during postnatal development. It is unclear how these molecules are assembled to form PNN around neurons, but some studies suggest that both neurons and glial cells are involved in the process (Miyata et al., 2007; Pyka et al., 2011). Astrocytes extend their processes into PNNs localized near the synapse (Brückner et al., 1993; Blümcke et al., 1995), suggesting a tetrapartite synaptic organization involving ECM, pre/post synaptic sites and astrocytes (Dityatev and Rusakov, 2011; Smith et al., 2015) whose synergistic functions remain unexplored.

## Parvalbumin Expressing Interneurons, PNNs and MMP-9

In the forebrain, PNNs are often found on PV expressing GABAergic neurons (**Table 1**). Other cell types, including putative excitatory neurons also contain PNNs but they are less numerous compared to GABAergic-PNN expressing cells (Wegner et al., 2003). Most studies detecting PNNs in the CNS use fluorescently coupled lectins, such as WFA and *vicia villosa agglutinin*, which bind *N*-acetylgalactosamine on chondroitin sulfate GAG side-chains of CSPGs (Nakagawa et al., 1986; Kosaka and Heizmann, 1989; Härtig et al., 1992). Immunolabeling using antibodies against specific PNN proteins, including aggrecan, tenascin and hyaluronan can also be used to visualize PNNs (Matthews et al., 2002; Dityatev et al., 2007; Giamanco et al., 2010; Yamada and Jinno, 2017). Studies comparing PNNs detected with lectins or specific antibodies indicate substantial levels of molecular heterogeneity (Matthews et al., 2002; Irvine and Kwok, 2018), highlighting the need for better approaches which address PNN heterogeneity (Irvine and Kwok, 2018). In the following discussion, most studies use lectin based detection of PNNs unless noted otherwise.

**Table 1:** Analysis of PV cells enwrapped with PNNs across sensory cortices.

	Mouse strain	Methods	PV cells with PNNs (%)
**Visual cortex**			
Pizzo et al., 2016	C57BL/6J	PV: (1) monoclonal mouse α-PV (SWANT, 1:500); (2) polyclonal guinea pig α-PV PNN: biotin-conjugated WFA (Sigma, 1:200)	All layers P18: ∼55% P35: ∼55%
Morishita et al., 2015	C57BL/6J	PV: monoclonal rabbit α-PV (SWANT, 1:500) PNN: biotin-conjugated WFA (Vector, 1:400)	All layers P50: ∼72%
Lensjø et al., 2017	C57BL/6J	PV: polycolonal rabbit α-PV (SWANT, 1:2000) PNN: biotin-conjugated WFA (Sigma, 1:200)	All layers 3–5 months:∼70%
Ueno et al., 2017c	C57BL/6J	PV: monoclonal mouse α-PV (Sigma, 1:1000) PNN: biotin-conjugated WFA (Vector, 1:200)	All layers 2–6 months: ∼50-60%
**Somatosensory cortex**			
Nowicka et al., 2009	Swiss albino mice	PV: (1) rabbit α-PV (1:1000); (2) monoclonal mouse α-PV (Sigma, 1:1000) PNN: biotin-conjugated WFA (Vector, 1:1000)	Layer 1–3 P20: <10% P30: ∼30% YA: ∼35% Layer 4 P10: ∼40% P20: ∼50% P30: ∼50% YA: ∼80% Layer 5–6 P10: ∼10% P30: ∼35% YA: ∼40%
Ueno et al., 2017b	C57BL/6J	PV: monoclonal mouse α-PV (Sigma, 1:1000) PNN: biotin-conjugated WFA (Vector, 1:200)	Layer 2-3 P14: ∼70%; P21, 28: ∼50% P56: ∼60% Layer 4 P14, 21, 28: ∼75% P56: ∼90% Layer 5-6 P14: ∼75% P21, 28: ∼40-50% P56: ∼60%
Karetko-Sysa et al., 2014	C57BL/6J	PV: polyclonal goat α-PV (Affinity BioReagents, 1:1000) PNN: biotin-conjugated WFA (Vector, 1:1000)	*^∗^Most PV cells colocalized with PNNs in layer 4* Layer 4-6 YA: 67%
**Auditory cortex**			
Brewton et al., 2016	C57BL/6J CBA/CaJ	PV: rabbit α-PV (SWANT, 1:10,000) PNN: FITC-conjugated WFA (Vector, 1:750)	All layers C57 YA: 35% CBA YA: 50%
Nguyen et al., 2017	CBA/CaJ	PV: rabbit α-PV (SWANT, 1:5000) PNN: FITC-conjugated WFA (Vector, 1:500)	All layers P30-60: 46%
Wen et al., 2017	FVB WT	PV: (1) rabbit α-PV (SWANT, 1:5000), (2) mouse α-PV (Sigma, 1:1000) PNN: FITC-conjugated WFA (Vector, 1:500)	Layer 2/3 P14: ∼1% P21: ∼29% P30: ∼15% Layer 4 P14: ∼18% P21: ∼38% P30: ∼38%
Ueno et al., 2017c	C57BL/6J	PV: monoclonal mouse α-PV (Sigma, 1:1000) PNN: biotin-conjugated WFA (Vector, 1:200)	All layers YA: ∼60-70%

*Note that these studies used WFA to stain for PNN. Layer-specific and age-related information are shown if available. P, postnatal day; Young adult (YA) = 3 months*.

Parvalbumin cells are a major class of GABAergic, fast-spiking inhibitory interneurons in the neocortex. Cortical PV cells contribute to synchronous oscillatory activity (Cardin et al., 2009; Sohal et al., 2009; Uhlhaas et al., 2009), particularly in the gamma range (∼30–100 Hz). Like PNNs, PV expression is developmentally regulated and experience-dependent (Hensch, 2005). In fact, PV and PNN colocalization is correlated with critical period closure and reduced synaptic plasticity, particularly in sensory cortices (Nowicka et al., 2009; Beurdeley et al., 2012). A large proportion of PNN-expressing PV cells is found within layer 4 of neocortex (**Table 1**). Interestingly, developing auditory cortex appears to exhibit fewer PNN and PV expressing cells than visual or somatosensory cortex, but in adult sensory cortices, a majority of PV cells express PNNs as seen with WFA staining. PNNs may regulate PV cell functions (Hensch, 2005; Cabungcal et al., 2013; Bernard and Prochiantz, 2016), as PNN reorganization is shown to result in abnormal CNS development and altered neural excitability (Pizzorusso et al., 2002; Dityatev et al., 2007; Liu et al., 2013; Balmer, 2016; Lensjø et al., 2017).

MMP-9 is a zinc-dependent endopeptidase expressed in various cells within the CNS which is capable of cleaving ECM proteins, including collagen and laminin (reviewed in Reinhard et al., 2015). Peak MMP-9 expression is observed during postnatal development and is reduced during adulthood (Bednarek et al., 2009; Reinhard et al., 2015; Wen et al., 2017). High levels of MMP-9 contribute to proteolytic cleavage of ECM, creating an extracellular environment that is permissive for synaptic plasticity. As aggrecan has been previously identified as an MMP-9 target (Mercuri et al., 2000; Madsen et al., 2010; Rankin-Gee et al., 2015), aggrecan-rich PNNs may be unstable in the presence of MMP-9, leading to abnormal development and neural excitability (Murase et al., 2017; Wen et al., 2017). A recent study by Murase and colleagues demonstrated that PV, PNN, and MMP-9 interactions are important for driving adult plasticity (Murase et al., 2017). In this study, monocular deprivation followed by light exposure in adult mice was associated with reduced WFA-expressing PNNs, lower aggrecan levels, and increased MMP-9 activity, as well as reduced PV cell output, increased firing of regular-spiking cells, and reduced gamma power (Murase et al., 2017). These findings suggest a link between PNNs, MMP-9 activity and PV activity that shapes normal brain development. Conversely, abnormal interactions may lead to neurological disorders.

## Fragile X Syndrome

Recently, we proposed a hypothesis for increased sensory hyperexcitability in Fragile X syndrome (FXS) through increased MMP-9 levels and abnormal PNN development (**Figure 1**). FXS is a leading genetic cause of autism, occurring in 1 in 4000 males and 1 in 8000 females (Murray et al., 1997). The most common cause of FXS is an expansion of CGG trinucleotide repeats in 5′ region of the *Fragile X mental retardation 1 (Fmr1)* gene that results in its hypermethylation and inactivation. This leads to loss of Fragile X Mental Retardation protein (FMRP), which is necessary for regulation of protein synthesis (Verkerk et al., 1991). Individuals with this neurodevelopmental disorder suffer from social and communication deficits, sensory hypersensitivity, and anxiety disorders (Abbeduto and Hagerman, 1997; Rogers et al., 2001). Auditory hypersensitivity is common in humans with FXS as measured by increased sound evoked responses, reduced habituation and expansion of brain regions activated by auditory stimuli (Rojas et al., 2001; Castrén et al., 2003; Van der Molen et al., 2012; Ethridge et al., 2016). These auditory processing deficits may drive social, cognitive and language delays and deficits in FXS (Rotschafer and Razak, 2014; Sinclair et al., 2017a,b).

Auditory hypersensitivity is also evident in *Fmr1* knockout (KO) mice, a mouse model of FXS, which exhibit increased susceptibility to audiogenic seizures (Musumeci et al., 2000) and abnormal auditory startle (Frankland et al., 2004). *In vivo* electrophysiological studies demonstrate that neurons in adult *Fmr1* KO mouse auditory cortex (1) are hyperexcitable, exhibiting increased responses per stimulus, (2) show more variable response latencies, and (3) display broader frequency tuning curves (Rotschafer and Razak, 2013). Recent work using EEG/ERP recordings shows that mature *Fmr1* KO mouse auditory cortex exhibits reduced habituation to repeated sound presentations (Lovelace et al., 2016). When resting EEG is analyzed for spectral power distribution, *Fmr1* KO mouse cortex shows increased gamma power. There is also enhanced N1 amplitude and reduced gamma phase locking in auditory ERPs (Lovelace et al., 2018). These phenotypes in the mouse model are remarkably similar to those seen in humans with FXS (Castrén et al., 2003; Ethridge et al., 2016; Wang et al., 2017). These results indicate that there is altered E/I balance in auditory cortex, which may underlie auditory hypersensitivity phenotypes. As PNNs are thought to be critical for maintaining E/I balance by modulating PV neuron activity, we tested the hypothesis that disruption of PNN development may underlie auditory hypersensitivity in FXS.

We found delayed PNN development and increased MMP-9 activity levels in the developing auditory cortex of *Fmr1* KO mice (Wen et al., 2017), which may lead to impaired PV cell development and reduced inhibition. These phenotypes were mostly observed between P14 and P21 suggesting a window of abnormal development that can be targeted for therapeutic applications. At P14, the responses of single neurons were similar across genotypes, while at P21, *Fmr1* KO neurons show hyper-responsiveness. Genetic reduction of MMP-9 levels in the *Fmr1* KO mice normalized PV/PNN development and also reversed the hyperresponsiveness of auditory cortical neurons. These findings suggest that MMP-9-mediated cleavage of PNNs during development may underlie sensory hypersensitivity in FXS. Reduction of MMP-9 levels with minocycline in humans with FXS (Dziembowska et al., 2013) contributes to reversal of several FXS phenotypes, including behavioral deficits and enhanced auditory electrocortical activity in adults and/or adolescents with FXS (Paribello et al., 2010; Leigh et al., 2013; Schneider et al., 2013). Minocycline administration in *Fmr1* KO mice prevents abnormal dendritic spine development in the hippocampus, improves anxiety-like behaviors (Bilousova et al., 2009; Dansie et al., 2013) and corrects ultrasonic vocalizations (Rotschafer et al., 2012). Genetic reduction of MMP-9 in *Fmr1* KO mice normalizes PNN formation and prevents the development of neuronal hyperexcitability (Lovelace et al., 2016; Wen et al., 2017), emphasizing the therapeutic potential of targeting MMP-9 and PNN in treatment of FXS.

In addition to regulating cortical development, MMP-9 cleavage of PNNs may drive changes in PV expression and/or PV cell functions seen in FXS. The somatosensory cortex of adult FXS mice exhibit delayed PV cell maturation (Nomura et al., 2017). In developing mice, PV interneurons receive reduced excitatory drive from neighboring pyramidal cells in layer 4 of somatosensory cortex (Gibson et al., 2008). PV cell maturation is characterized by gradual increases in signaling, including faster firing rates and IPSC kinetics (Lazarus and Huang, 2011). An orthodenticle homeoprotein, Otx2, may play a role in PV cell maturation and refinement of cortical inhibition. Otx2 expression coincides with both PV and PNN expression in the cortex during critical period plasticity (Sugiyama et al., 2008). Studies of rodent visual cortex development indicate that Otx2 is localized to GABAergic cells, and is more likely found in PV cells that are enwrapped with PNNs, which contain a specific Otx2 binding site (Beurdeley et al., 2012). PNNs are necessary for the uptake of Otx2 by PV cells, which promotes PV cell maturation (Beurdeley et al., 2012). RNA sequencing analysis of PV cells containing Otx2 indicates that Otx2 may be necessary for mitochondrial function, cellular respiration, and redox regulation (Sakai et al., 2017). These data suggest that PNNs may drive PV cell maturation by directing transcription or translation of specific genes necessary for PV cell function. In FXS, delayed PNN expression around PV cells during development may contribute to abnormal PV cell maturation.

Network excitability in FXS may also result from impaired synaptic scaling at PV cells due to PNN dysfunction. There is evidence for a role of PNNs in mediating synaptic scaling via neuronal activity-regulated pentraxin, also known as *Narp*. *Narp* can interact with the N-terminal domain of AMPA receptors localized extracellularly and facilitate AMPA receptor clustering at both the pre- and post-synaptic cell in response to stimulation (O’Brien et al., 1999). Studies by Chang et al. (2011) in developing mouse hippocampus demonstrated that (1) *Narp* was highly expressed at excitatory synapses on PV interneurons, (2) PNN expression facilitated *Narp* expression (and conversely PNN degradation contributed to reduced *Narp* levels), and (3) *Narp* expression at PV interneurons was necessary for increased AMPA receptor-mediated mEPSC amplitude in response to stimulation both *in vitro* and *in vivo*. This suggests that during development, sensory stimuli can activate *Narp* expression which promotes PV interneuron excitation, leading to feed-forward inhibition (O’Brien et al., 1999; Gu et al., 2013). In FXS, delayed development of PNNs may contribute to abnormal *Narp* expression, impairing the ability of PV cells to scale cortical networks, resulting in cortical hyperexcitability.

## Schizophrenia

One mechanism that has been proposed to explain some symptoms of schizophrenia is a reduction in GABAergic signaling in specific cortical regions. This is evident from a number of studies characterizing GABAergic cells using antibodies which target GABA-synthesizing enzymes glutamate decarboxylase 65 and 67 (GAD65 and GAD67) in brain tissues from schizophrenia patients. *In situ* hybridization and RT-PCR studies indicate that post-mortem brain tissues from schizophrenia patients exhibit reduced PV and/or GAD67 mRNA, particularly in prefrontal cortex (Akbarian et al., 1995; Volk et al., 2000; Hashimoto et al., 2003, 2008a,b; Mellios et al., 2009). Reduced PV and GAD67 protein levels are also seen in both prefrontal and temporal cortex of post-mortem brain tissues (Beasley and Reynolds, 1997; Curley et al., 2011; Glausier et al., 2014). Cortical gamma band oscillations have been attributed to GABAergic cell activity, in particular PV interneurons (Sohal et al., 2009). Increased gamma-band power was observed in baseline EEG recordings from humans with schizophrenia (Gandal et al., 2012). In contrast, gamma-band activity that is driven by rapid presentation of auditory stimuli and synchrony or phase-locking of electrocortical activity to these stimuli are reduced in schizophrenic patients compared to healthy controls (Kwon et al., 1999; Light et al., 2006). These data suggest altered PV cell function in schizophrenia, but why these cells are susceptible is unclear.

Redox dysregulation has been observed in schizophrenic patients, who have defective antioxidant systems and exhibit increased lipid peroxidation (Do et al., 2000; Yao et al., 2006; Flatow et al., 2013). Genetic analysis of single nucleotide polymorphisms in schizophrenia patients demonstrates mutations in the gene encoding GCL, an enzyme that is important for the production of the cellular antioxidant GSH (Tosic et al., 2006). PV cells are particularly susceptible to oxidative stress given their fast cellular metabolism and increased mitochondrial function (Hasenstaub et al., 2010; Kann et al., 2014; Inan et al., 2016). Recent studies show that PNNs may protect PV cells from oxidative stress (Cabungcal et al., 2013; Morishita et al., 2015), and thus serve as a ‘safety net’ to support the rapid firing properties of PV cells. Cabungcal and colleagues analyzed the levels of oxidative stress in PV cells with and without PNNs in GCL KO mice, a mouse model of schizophrenia, by measuring the levels of 8-hydroxy 2 deoxyguanosine (8-oxo-dG). During development, GCL KO mice had fewer PNN-expressing PV cells in anterior cingulate cortex, and the injection of a dopamine reuptake inhibitor to promote more oxidative stress via buildup of reactive oxygen species contributed to increased 8-oxo-dG signal and PV cell loss. Further analysis indicated that while PV neurons with PNNs were less susceptible to oxidative stress, chABC-mediated degradation of PNNs made PV cells more vulnerable (Cabungcal et al., 2013). As chABC removes GAG side chains from CSPGs (Bradbury et al., 2002), these results suggest that the neuroprotective effects of PNNs may be attributed to the CS-side chains of CSPGs. Specifically, *in vitro* studies conducted in human neuroblastoma SH-SY5Y cells indicate that chondroitin sulfate application prior to exposure to oxidative-stress inducing agents, such as hydrogen peroxide, can prevent cell death and attenuate the production of reactive oxygen species. Analysis of molecular mechanisms indicates that chondroitin sulfate promotes activation of protein kinase pathways including PKC and PI3K/Akt, and subsequent production of a neuronal antioxidant, heme oxygenase-1 (Cañas et al., 2007).

Perineuronal net dysfunction in schizophrenia may also drive changes in GABAergic cell function leading to network dysfunction, as normal PNN expression regulates excitability of PV cells (Liu et al., 2013; Balmer, 2016). Härtig et al. (1999) speculated that the large, negatively charged sugar chains, which are enriched in PNNs, might allow for buffering of sodium and potassium ions at synapses. While this mechanism has not been confirmed, chABC-mediated removal of chondroitin sulfate side chains from PNNs reduced spiking of PV cells in adult rats (Balmer, 2016; Lensjø et al., 2017) and increased spiking variability (Lensjø et al., 2017). In addition, brevican conditional knockdown in PV-expressing cells demonstrated that changes in the expression of specific lecticans alter synaptic input and firing frequency in PV cells (Favuzzi et al., 2017). These findings lend support to the idea that PNN composition plays an important role in regulating PV cell function.

As PNNs regulate GABAergic cell function and oxidative stress levels, cortical abnormalities observed in schizophrenia may be attributed to PNN dysfunction (reviewed in Berretta, 2012; Berretta et al., 2015). Indeed, several studies indicate that schizophrenia is associated with the loss of PNNs. Post-mortem brains from patients with schizophrenia exhibit reduced density of WFA-labeled cells (Mauney et al., 2013) and reduced levels of CSPGs and PV expression in prefrontal cortex (Enwright et al., 2016). A mouse model of schizophrenia also exhibits reduced PNN levels in prefrontal cortex (Cabungcal et al., 2014). In this model, schizophrenia-like abnormalities are reported in the adult mouse prefrontal cortex as a result of neonatal lesion of ventral hippocampus. The effects of dysregulated dopamine signaling on prefrontal cortex oscillatory activity, which are suggested to drive schizophrenia phenotypes, can be also regulated by PNNs. For example, network oscillatory activity that was increased by drug-induced changes in dopaminergic signaling in mouse prefrontal cortex was further enhanced following chABC-mediated degradation of PNNs (Steullet et al., 2014).

Recent studies have hinted at the potential role of MMP-9 in the manifestation of schizophrenia phenotypes. A functional polymorphism of the MMP-9 gene was discovered in human schizophrenia patients (Rybakowski et al., 2009; Lepeta et al., 2017). Additionally, Yamamori et al. (2013) showed elevated MMP-9 levels in the plasma of schizophrenic patients. Various clinical trials have been conducted using minocycline alone or as an adjunct to risperidone in patients with schizophrenia and have demonstrated overall improvement in negative symptoms and executive functioning (Miyaoka et al., 2008; Levkovitz et al., 2010; Khodaie-Ardakani et al., 2014). Beside its antibiotic activity, minocycline can reduce plasma levels of MMP-9 (Dziembowska et al., 2013) and inhibit MMP-9 activity (Lee et al., 2006), suggesting a possible role of MMP-9 inhibition in the beneficial effects of minocycline in schizophrenia. Clearly additional studies are needed to test whether elevated MMP-9 levels do indeed drive PNN changes observed in prefrontal cortex of schizophrenia mouse models and how it relates to the human condition. Future studies can help to determine whether ECM abnormalities underlie the changes in GABAergic signaling and redox dysregulation associated with schizophrenia.

## Seizures and Epilepsy

Epilepsy occurs unpredictably and affects people of all ages, although onset commonly occurs in childhood (Hauser, 1992). It is idiopathic and can include generalized seizures, which involve epileptiform activity emanating from the entire cortex, as well as focal or partial seizures, which result from epileptiform activity from a single part of the cortex (Hauser, 1992). The molecular mechanisms underlying seizure onset include impaired PV cell functions. Analysis of post-mortem human brain tissues demonstrated reduced PV cell density and immunoreactivity at epileptic foci in the hippocampus and neocortex (Sloviter et al., 1991; Ferrer et al., 1994; Andrioli et al., 2007). Genomic sequencing data indicate that a number of mutations in the *Scn1a* gene contribute to a spectrum of different epileptic disorders affecting children, including *de novo* (Claes et al., 2001; Fujiwara et al., 2003) as well as missense, frame-shift, and nonsense mutations (Fujiwara et al., 2003). *Scn1a* is a gene that encodes Nav1.1, a sodium channel necessary for PV cell firing (Ogiwara et al., 2007; Hedrich et al., 2014). Recent studies demonstrate that reduced PV cell spike output may contribute to seizures in mice with genetic knock-in of mutated human *Scn1a*, which results in subtle changes in Nav1.1 functionality (Ogiwara et al., 2007; Hedrich et al., 2014). Several rodent models of epilepsy, which use drugs to induce seizures, also shed some light on the role of PV cells. For example, PV KO mice are more likely to experience status epilepticus compared to controls following increased doses of pentylenetetrazol (PTZ), a GABA_A_ receptor blocker (Schwaller et al., 2004).

As PNNs regulate PV cell activity (Balmer, 2016; Lensjø et al., 2017) and protect PV cells from oxidative stress (Cabungcal et al., 2013), PNNs can be potentially targeted to control seizures. Recent work has reported changes in PNN integrity in epilepsy. Drug-induced seizures led to increased expression of aggrecan cleavage products around PV cells and reduced expression of PNN components in rodent hippocampus, including Ctrl1 and hyaluronan synthase 3, both of which help to stabilize PNNs (McRae and Porter, 2012; Rankin-Gee et al., 2015). Drug-induced seizures increased unbound CSPG levels and decreased the levels of CSPGs associated with PNNs around PV cells, which were detected with WFA in cerebral cortex (Yutsudo and Kitagawa, 2015). In rodent piriform cortex, kindling-induced seizures caused PNN degradation and increased GABAergic synapses, contributing to the rewiring of cortical networks (Pollock et al., 2014). Moreover, PNN breakdown induced by seizures was at least partially facilitated by increased enzymatic activity of MMPs (Kim et al., 2009; Rankin-Gee et al., 2015), and seizure susceptibility was reduced in MMP-9 KO mice (Wilczynski et al., 2008). This suggests that PNN breakdown via MMP-9 following seizure induction contributes to network rewiring, leading to pathological hyperexcitability. Future studies should explore this PNN loss-hyperexcitability connection further and determine whether there would be therapeutic benefit to MMP inhibition and PNN restoration in epilepsy.

## CNS Injury and Stroke

In CNS injury and stroke, re-establishing neuronal plasticity appears to be key for re-innervation and recovery. A full summary of this literature is outside the scope this review, so only a few exemplars are mentioned to make the point that understanding the contributions of PNN and other ECM molecules may be critical in developing treatments, because the CSPGs present in PNNs appear to inhibit axonal growth (Kwok et al., 2011). In rat spinal cord injury models, increased concentrations of CSPGs and PNN expression were found at injury sites and surrounding secondary dorsal column relay neurons (Massey et al., 2008). When sensory axons were transplanted into adult rats with dorsal column lesions, neurons were able to sprout and extend their processes to additional denervated areas, but failed to penetrate the original lesion sites, which lead to denervation of upstream pathways and inactivation of parts of somatosensory cortex (Davies et al., 1999; Kaas et al., 2008). CSPG degradation via application of chABC promoted regeneration of dorsal column axons after injury. This not only resulted in an increase in terminals from initially injured afferents, but also contributed to expanded receptive fields of uninjured primary afferents (Bradbury et al., 2002; Massey et al., 2006).

Studies in stroke models indicate that injury onset leads to two processes. On the one hand, focal cortical stroke in rats contributes to upregulation of a number of CSPG-encoding genes particularly near the infarct and glial scar. RT-PCR analysis of the affected tissue indicated upregulation of neurocan, which peaked on the third day following middle cerebral artery occlusion, as well as versican and brevican, both of which peaked by day 28 (Carmichael et al., 2005). In contrast, in cortical areas surrounding the infarct and outside of the glial scar, WFA-expressing PNNs are completely degraded by day 7, and CSPG immunoreactivity is down regulated although they remain detectable up to 14 days post-injury (Hobohm et al., 2005). Similar degradation of WFA-expressing PNNs in the neocortex has also been observed following thromboembolic middle cerebral artery occlusion in rats, permanent middle artery occlusion in mice, and focal cerebral ischemia in sheep (Härtig et al., 2017). Interestingly, reduced PV-immunoreactivity and PV cell function have also been observed following stroke and this decrease in inhibitory cell function may underlie epilepsy-like activity following stroke (Hobohm et al., 2005; Xie et al., 2014). As CSPGs inhibit growth, these findings indicate that spinal cord injury, stroke and/or traumatic brain injury may contribute to ECM reorganization, allowing neuronal growth and plasticity in areas free from damage and inhibiting growth where injury-induced cellular damage is more severe (Hobohm et al., 2005; Harris et al., 2010). MMP-9 may be responsible for this ECM reorganization as a number of studies have demonstrated increased MMP-9 levels following CNS injury and stroke in humans as well as animal models (Zhang et al., 2011; Chaturvedi and Kaczmarek, 2014; Liu et al., 2014).

## Alzheimer’S Disease

Alzheimer’s disease is associated with memory impairment, which may be attributed to intracellular buildup of neurofibrillary tangles and the accumulation of extracellular beta-amyloid plaques in hippocampus and cortex. This may lead to hippocampal and cortical hyperexcitability as well as increased seizure susceptibility in both AD humans and rodent models (Palop et al., 2007; Roberson et al., 2011). Post-mortem brains of AD patients exhibit reduced expression of PNNs, particularly in the hippocampus (Baig et al., 2005; Lendvai et al., 2013). Neurons that exhibit buildup of tau protein and neurofibrillary tangles are more likely to lack PNNs (Brückner et al., 1999; Morawski et al., 2010). Direct support for the neuroprotective effects of PNNs from AD-related pathology was demonstrated in rodent cortical neurons *in vitro.* These experiments showed that PNN-ensheathed cells were more resistant to cell death when neurons were incubated with exogenous monomeric Aβ1-42 peptide for 48 h. More cells died when pretreated with chABC (Miyata et al., 2007). The neuroprotective abilities of PNNs may stem from their ability to act as a barrier for oxidative stress-inducing molecules, such as metal ions. The molecules can react with free radicals generated by cellular metabolism to generate even more free radicals, resulting in lipid peroxidation, oxidative stress, and cell death (Stohs and Bagchi, 1995). Indeed, cortical cells expressing PNNs exhibited accumulation of iron in post-fixed brain tissues obtained from both AD and normally aged controls (Reinart et al., 2003). Accumulation of lipofuscin, a byproduct of iron-catalyzed oxidative stress, was also observed in cells without PNN (Morawski et al., 2004). These results indicate that cells without PNNs are more susceptible to cell death (Morawski et al., 2004). Similar results have been obtained in AD rodent models, which rely on bilateral or unilateral microinjection of iron chloride into mouse cortex to mimic AD-related oxidative stress. While iron-induced oxidative stress resulted in apoptotic cell death in cells without PNNs, neurons with PNNs were more likely to survive (Suttkus et al., 2012, 2014). Genetic reduction of aggrecan, which has the highest number of CS-side chains and presumably the largest negative charge, resulted in reduced neuroprotective effects (Suttkus et al., 2014). These results lend further support to the idea that the CS-side chains of PNNs mediate its neuroprotective effects.

Perineuronal net loss in AD may contribute to altered E/I balance (Palop et al., 2007; Sun et al., 2009), synaptic loss (Scheibel and Tomiyasu, 1978; Ruiter and Uyling, 1987; Hoover et al., 2010) and increased susceptibility to oxidative stress (Deibel et al., 1996; Varadarajan et al., 2000; Manczak et al., 2006; De Felice et al., 2007). Interestingly, some studies suggest that MMP-9 may be a key molecular target in treating AD, as MMP-9 is capable of cleaving Aβ *in vitro* (Backstrom et al., 1996), and plasma levels of MMP-9 are elevated in AD patients (Backstrom et al., 1996; Lorenzl et al., 2003). As MMP-9 is also involved in PNN maintenance, elevated MMP-9 levels may contribute to both PNN degradation and altered levels of Aβ in AD.

## Hearing Loss and Central Auditory Processing

Recent studies suggest that loss of PNNs around PV cells occurs following noise-induced and age-related hearing loss. Overexposure to loud sounds can cause hair cell damage (Puel et al., 1998), hearing loss and reduced afferent input in the auditory system (Syka, 2002). Noise-induced hearing loss leads to increased excitability (gain) in the central auditory system, but the cellular mechanisms are not clear. PNNs are impaired following noise induced hearing loss, even within 1 day after noise exposure. Specifically, PNN intensity is reduced in layers 1–4 of auditory cortex of adult CBA mice 1, 10, and 30 days post-noise exposure. In layers 5–6, PNN intensity decreases 10 days post-noise exposure (Nguyen et al., 2017). Reduced PNN intensity may be a result of increased cleavage or degradation of various PNN components, such as CSPGs, hyaluronan, or link proteins. In brainstem auditory nuclei of mice, genetic loss of brevican contributes to loss of brevican-expressing PNNs as well as reduced sound-evoked firing rates and increased neuronal response thresholds (Blosa et al., 2015). chABC-mediated degradation of PNNs around PV cells in brainstem and central auditory neurons has similar effects, resulting in reduced PV cell excitability and gain (Balmer, 2016). These results indicate that PNNs may be necessary for neuronal excitability throughout the auditory pathway. Understanding the auditory system’s ability to control excitability as a result of exposure to traumatic sounds and the potential role of PNNs in regulating this process may help to characterize molecular targets in treatment of noise induced hearing loss and tinnitus.

Age-related hearing loss, also known as presbycusis, is associated with difficulty interpreting speech and slower auditory processing (Gates and Mills, 2005) which may lead to social isolation and cognitive decline. Hearing aids amplify sounds, but may not improve speech processing, suggesting central auditory system changes. Neuronal response properties susceptible to presbycusis include frequency selectivity, spontaneous activity levels and spectrotemporal selectivity (Trujillo and Razak, 2013). GABAergic signaling is involved in shaping these properties, with fast-spiking PV cells shaping both spectral and temporal selectivity (Moore and Wehr, 2012). Indeed, several studies have demonstrated reduced GABAergic signaling in the aged auditory system of rodents and humans (Caspary et al., 1990; Ouda et al., 2008; Martin del Campo et al., 2012; Gao et al., 2015).

Perineuronal nets may play a major role in PV neuron susceptibility and altered cortical processing in presbycusis. The number of PNN expressing cells is reduced in aging mouse auditory cortex with a particular impact on PV/PNN co-localized cells (Brewton et al., 2016). As PNNs protect PV cells from oxidative stress, these data indicate that PNN downregulation with age may make PV cells more susceptible to cell death. Altered PNN formation may contribute to abnormal GABAergic signaling and impaired temporal processing with age, leading to deficits in speech comprehension. Additional studies are required to identify the specific components of PNNs that are altered in noise- and age-related hearing loss.

## Conclusion and Future Studies

Accumulating evidence suggests that PNN loss is associated with a number of neurological disorders. PNNs are also implicated in experience-dependent plasticity during development. In fact, activity-dependent synaptogenesis and the development of stable neuronal networks may be a result of normal PNN interactions with inhibitory interneurons, in particular fast spiking PV cells. Despite the evidence for developmental and pathological changes in PNNs, their precise role in network function and development is not known. Additional studies are needed that compare intrinsic and synaptic properties of the same cell types with and without PNNs. While the use of chABC has been useful in addressing PNN function, this enzyme likely has broader effects. Additional methods such as genetic removal of specific PNN components are needed.

Considering the prominent role of PNNs in many processes within the CNS, more focus should be placed on characterizing the mechanisms of PNN regulation in the healthy and diseased brain. Some of the work reviewed here indicates that PNNs are necessary for PV cell function, yet in several brain areas not all PV cells are ensheathed by PNNs as labeled with WFA (**Table 1**). PNNs are heterogeneous structures (Ueno et al., 2017b; Yamada and Jinno, 2017; Irvine and Kwok, 2018), and PNN composition may be brain region- and developmental age-specific. For instance, in the rodent hippocampus, aggrecan-expressing PNNs overlap with WFA labeling in stratum oriens, but stratum pyramidale exhibits aggrecan-rich PNNs, which are not WFA-positive (Yamada and Jinno, 2017). PNNs containing aggrecan and brevican do not always overlap in mouse somatosensory cortex (Galtrey et al., 2008; Ueno et al., 2017b). Tenascin-R expressing PNNs are not observed in rodent PFC, but are prominent in other cortical areas (Ueno et al., 2017a). These findings suggest that future work characterizing other PNN components such as hyaluronan (Oohashi et al., 2015) will be helpful in understanding PNN development and re-organization in health and disease. New models that allow for the examination of PNN formation and restructuring in the living brain will contribute to a better understanding of its role in circuit function. Genetic approaches to visualize and disassemble PNNs will also be very important moving forward.

Mechanisms of PNN loss and restructuring in disease are still not clear. One of the ECM modifying enzymes, MMP-9, has been hypothesized to play a role in pathophysiology of schizophrenia, AD and FXS. As MMP-9 is capable of cleaving ECM, elevated MMP-9 levels can drive PNN loss and re-organization. As there is evidence for abnormal MMP-9 regulation in FXS, schizophrenia and AD as well as altered PNNs in presbycusis and acoustic trauma models, studies of how MMP-9 is involved in PNN regulation are crucial for the development and validation of future therapeutics. Recent studies indicate that minocycline administration may reverse both schizophrenia and FXS phenotypes, perhaps due to its ability to lower MMP-9 levels or activity (Dziembowska et al., 2013). As MMP-9 cleaves PNN components, perhaps the use of more specific MMP-9 inhibitors may yield higher efficacy in treatment of these and other neurological disorders.

While we have focused on the potential role of MMP-9 in regulating PNNs and neural excitability in disease pathology, the ADAMTs family of aggrecanases may also cause PNN cleavage and reorganization. Indeed, several ADAMTs are upregulated following spinal cord injury, stroke, and epilepsy (Lemarchant et al., 2013; Kim et al., 2016). In addition, a number of molecules can regulate MMP-9 activity itself, including tissue inhibitors of MMPs, known as TIMPs, which form 1:1 complexes with MMPs in order to inhibit MMP activity (Gomis-Rüth et al., 1997; Brew and Nagase, 2010). TIMP levels are increased in postmortem brains of AD patients (Peress et al., 1995), and may be indicators for risk of stroke mortality (Hansson et al., 2011). Schizophrenia is associated with higher MMP-9/TIMP1 ratios compared to healthy controls (Rahimi et al., 2017). Future work characterizing the expression of other MMPs in disease pathology may also be useful as a number of MMPs can directly or indirectly activate MMP-9, including MMP-7, MMP-3, and MMP-14. Quantitative PCR analysis indicates that transcripts from MMP-9, 3, 7, and 14 are upregulated in rodent models of spinal cord injury (Zhang et al., 2011). Understanding how other MMP-9 and PNN modifying enzymes are regulated may contribute to the development of new targets in treating neurological disorders.

As PNNs may play a major role in disease pathology, the development of therapeutic interventions, which target these structures, may be key in restoring neuronal excitability and cortical function. For instance, recent findings in rodent stroke models indicate that rats housed in enriched environments following ischemic damage exhibited faster neurological recovery compared to rats placed in standard housing (Madinier et al., 2014; Quattromani et al., 2018). Quatrromani and colleagues suggest that this phenomenon may be attributed to sensorimotor stimulation which results in increased protease activity and reduced expression of PNNs. As the absence of PNNs is associated with increased plasticity (Pizzorusso et al., 2002; Nowicka et al., 2009), this may allow for axonal outgrowth and synaptogenesis, particularly in surviving cortical areas, which exhibit reduced CSPG and PNN expression and are therefore growth permissive (2014). These findings suggest that comparable human treatments, which target PNNs specifically, might involve non-invasive brain stimulation or combination brain stimulation/protease inhibitor treatments. While brain stimulation techniques are already currently utilized to treat neurological disorders (Cirillo et al., 2017), understanding how PNNs are modified following stimulation in rodents would lend support for use of this treatment paradigm, particularly in disorders involving abnormal PNNs.

While we do not emphasize inflammatory response pathways in the present review, these may be crucial in understanding MMP-9 dysregulation in CNS disorders as there is evidence for neuro-inflammation in injury models, AD, epilepsy, and schizophrenia (Papa et al., 2014). Cirillo and colleagues suggest that following insult, reactive gliosis triggers activation of MMPs and maladaptive plasticity, perhaps due to increased degradation of neurotrophins such as NGF (De Luca et al., 2016; Cirillo et al., 2016). Neurotrophins are necessary for neuronal growth and plasticity, and increased MMP-9 activity has been shown to degrade mature NGF (Bruno and Cuello, 2006). Moreover, recent work in peripheral nerve injury models indicates that co-treatment with MMP-9 inhibitors and NGF peptide following insult can restore synaptic homeostasis, i.e., normalize glutamate/GABA ratio and expression of glial amino acid transporters (De Luca et al., 2016; Cirillo et al., 2016). Unlike FXS, where MMP-9 upregulation can likely be attributed to loss of the *Fmr1* gene (Janusz et al., 2013), these findings provide important mechanistic insight for how MMP-9 dysregulation might arise in other CNS diseases. Whether NGF downregulation alters PNN expression remains to be studied. However, if NGF does play a role, this has important implications for the development of new PNN-targeting therapies.

## Author Contributions

TW reviewed the literature and wrote the manuscript. IE, DB, and KR were involved in writing and editing the manuscript.

## Conflict of Interest Statement

The authors declare that the research was conducted in the absence of any commercial or financial relationships that could be construed as a potential conflict of interest. The reviewer GC and handling Editor declared their shared affiliation.

## Abbreviations

AD: Alzheimer’s disease
ADAMTs: a disintegrin and metalloproteinase with thrombospondin motif
chABC: chondroitinase ABC
CNS: central nervous system
CSPG: chondroitin sulfate proteoglycan
E: embryonic
E/I: excitatory/inhibitory
ECM: extracellular matrix
EEG: electroencephalography
FXS: Fragile X Syndrome
GAD: glutamate decarboxylase
GAG: glycosaminoglycan
GCL: glutamate cysteine ligase
GSH: glutathione
HAS: hyaluronan synthase
MMP-9: matrix metalloproteinase-9
NGF: nerve growth factor
Otx2: orthodenticle homeoprotein 2
P: postnatal
PNN: perineuronal net
PV: parvalbumin
TIMPs: tissue inhibitor of MMPs
WFA: wisteria floribunda agglutinin.
